# Infiltration der Schädelkalotte durch ein dedifferenziertes kutanes Leiomyosarkom

**DOI:** 10.1007/s00105-021-04800-w

**Published:** 2021-03-24

**Authors:** J. C. Maier, K. Kofler, S. Forchhammer, M. Skardelly, T. Mentzel, H. M. Häfner, L. Kofler

**Affiliations:** 1 Universitätshautklinik Tübingen, Liebermeisterstr. 25, 72076 Tübingen, Deutschland; 2 Praxis für Neurochirurgie Reutlingen, Steinenbergstr. 31, 72764 Reutlingen, Deutschland; 3 Dermatopathologie Friedrichshafen, Siemensstr. 6/1, 88048 Friedrichshafen, Deutschland

**Keywords:** Dermatochirurgie, 3‑D-Histologie, Dermatoonkologie, Metastasierungspotenzial, Schnittrandkontrollierte Resektion, Dermatosurgery, Three-dimensional histology, Dermato-oncology, Metastasis risk, Margins of excision

## Abstract

Wir berichten über einen 81-jährigen Patienten mit bereits mehrfach voroperiertem Tumor der Kopfhaut. In der histologischen erneuten Aufarbeitung ließ sich ein dedifferenziertes Leiomyosarkom nachweisen, das bereits die Schädeldecke infiltriert hatte. In einem interdisziplinären Ansatz mit den Kollegen der Klinik für Neurochirurgie konnte eine Komplettexzision erreicht werden. Kutane Leiomyosarkome sind seltene Tumoren der Haut, die typischerweise als langsam wachsende, erythematöse Knoten imponieren. Aufgrund des Metastasierungspotenzials ist eine komplette, schnittrandkontrollierte Resektion erforderlich.

## Abstract (en)

We present a brief report of an 81-year-old man with a pretreated leiomyosarcoma of the skull. Histologically the diagnosis of a dedifferentiated cutaneous leiomyosarcoma with an infiltration of the skull was confirmed. In an interdisciplinary approach together with the University Clinic for Neurosurgery, complete removal of the tumour was performed. Cutaneous leiomyosarcoma are rare tumors of the skin and typically present as slowly growing erythematosus nodes. Because of the risk of metastatic spread, complete micrographically confirmed resection is necessary.

Wir berichten über den ungewöhnlichen Verlauf eines 81-jährigen Patienten, der sich in unserer Klinik mit dem Rezidiv eines extern bereits mehrfach voroperierten Tumors am Kapillitium vorstellte.

## Anamnese

Ein 81-jähriger Patient stellte sich erstmalig in unserer Klinik bei wachsendem rötlichem Knoten am Kapillitium vor. In der Vorgeschichte wurden aufgrund eines rezidivierenden dermalen Sarkoms mehrmalige chirurgische Resektionen an der Kopfhaut durchgeführt.

Nebendiagnostisch waren eine koronare Herzerkrankung mit stattgehabtem Myokardinfarkt und erfolgter Stenteinlage sowie eine arterielle Hypertonie bekannt.

## Klinischer Befund

**Figure 1:**
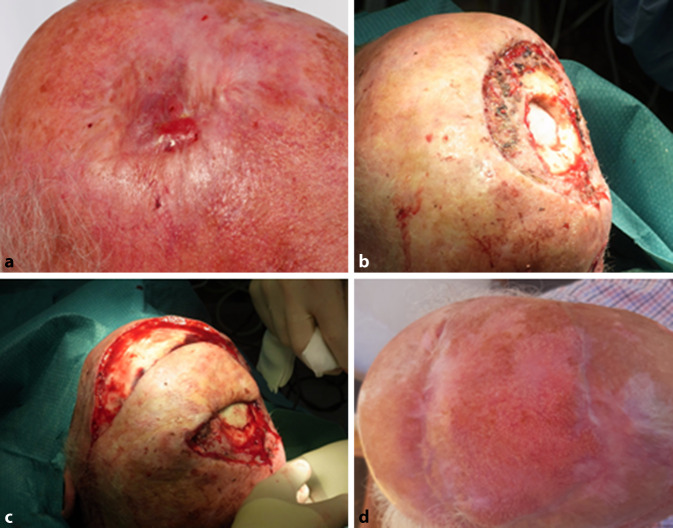

Klinisch zeigte sich bei Erstvorstellung eine flächige Narbenplatte von etwa 40 ×50 mm bei extern mehrfachen Voroperationen. Im Randbereich fiel ein derber, erythematöser, nicht verschieblicher, teils erosiver Knoten auf (Abb. 1a). Präoperativ konnten eine lokoregionäre Metastasierung bzw. Fernmetastasen mittels Lymphknotensonographie sowie Computertomographie ausgeschlossen werden.

## Diagnose

**Figure 2:**
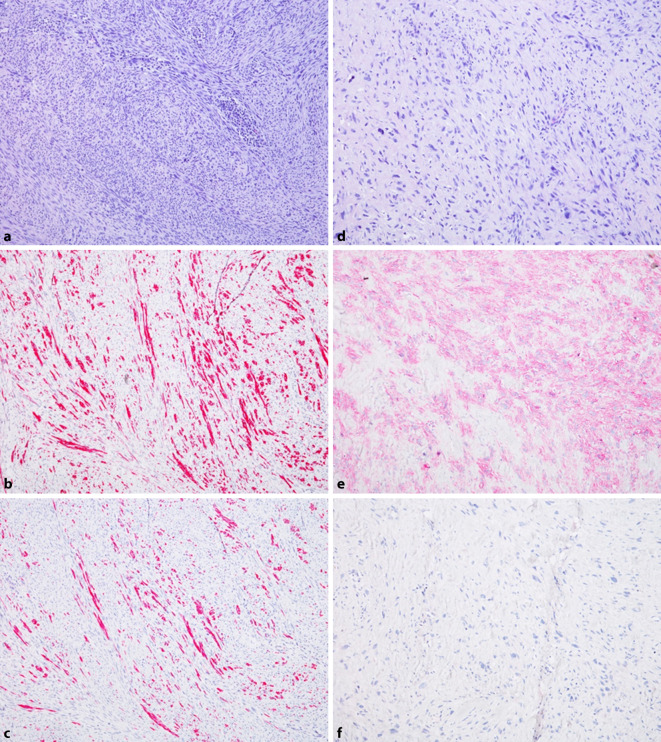

In der histologischen Aufarbeitung der vorliegenden Präparate zeigten sich bereits HE(Hämatoxylin-Eosin)-morphologisch 2 distinkte Tumorkomponenten. Zum einen zeigt sich eine spindelzellige, faszikulär wachsende Komponente mit stumpfen Zellkernen und glattmuskulärer Differenzierung. Diese zeigt immunhistochemisch Positivität gegenüber sm-Aktin, Calponin, h‑Caldesmon und Desmin (Abb. 2a–c). Darüber hinaus zeigt der Großteil der Tumorzellen eine pleomorphe, entdifferenzierte Morphologie. Hier finden sich atypisch vergrößerte, teils hyperchromatische Tumorzellen mit flauer immunhistochemischer Expression von sm-Aktin, jedoch Negativität für h‑Caldesmon und Desmin (Abb. 2d–f).

In der Zusammenschau der Befunde wurde die Diagnose eines dedifferenzierten Leiomyosarkoms gestellt.

## Therapie und Verlauf

Wir führten eine 3‑D-histologisch kontrollierte Exzision des Tumors durch, wobei eine Infiltration bis in die Schädelkalotte nachgewiesen werden konnte. Eine Bildgebung mittels Computertomographie wurde daraufhin ergänzt, um das Ausmaß der knöchernen Beteiligung präoperativ darzustellen. In einem interdisziplinären Ansatz erfolgten gemeinsam mit den Kollegen der Neurochirurgie die Trepanation und vollständige Resektion des Knochens unter Schonung der Dura mater (Abb. 1b). Anschließend wurde der knöcherne Defekt mittels Palacos® (Heraeus Medical GmbH, Wehrheim) verschlossen. Aufgrund der Knochenabtragung mit einer Größe von 5,5×5 cm wurde eine Deckung mit Palacos® durch den Neurochirurgen indiziert. Eine Transpositionslappenplastik wurde zur Deckung durchgeführt (Abb. 1c). Diese war notwendig, um den durch Palacos® verschlossenen Knochendefekt zu decken. Die Lappenversorgung wurde durch die breite Lappenbasis sichergestellt. Als alternatives Deckungsverfahren wäre prinzipiell auch ein mikrovaskulär gestielter Lappen denkbar, der jedoch sowohl chirurgisch als auch in der Einheilung als komplexer anzusehen ist. Die postoperative Wundversorgung erfolgte durch tägliche Verbände mittels Jelonet® (Smith & Nephew GmbH, Hamburg) und Kompressenverband. Bis zur vollständigen Einheilung vergingen 7 Wochen.

In der 3‑D-histologischen Aufarbeitung zeigten sich tumorfreie Basis- und Randschnitte, bezüglich des ossären Resektionsstatus konnte eine R0-Resektion erzielt werden. Insgesamt zeigte sich ein Sicherheitsabstand von 5 mm.

Der Patient wurde in unsere Nachsorgesprechstunde eingeschlossen, in der regelmäßige klinische und sonographische Kontrollen erfolgen. Die Transpositionslappenplastik zeigte sich im Verlauf vollständig eingeheilt (Abb. 1d). Aktuell besteht 22 Monate postoperativ klinisch und sonographisch kein Hinweis auf ein Rezidiv.

Die durchgeführte Transpositionslappenplastik birgt die Gefahr, dass Rezidive spät erkannt werden können. Es sind somit engmaschige Kontrollen sowie regelmäßige Bildgebungen mittels Schädelcomputertomographie zur frühzeitigen Erkennung möglicher Rezidive notwendig.

## Diskussion

Das kutane Leiomyosarkom ist ein seltener Weichteiltumor der Haut [1], wobei der Erkrankungsgipfel zwischen dem 6. und 7. Lebensjahrzehnt liegt. Prinzipiell unterscheidet man das dermale Leiomyosarkom vom subkutanen Leiomyosarkom [2]. Klinisch sind kutane Leiomyosarkome durch langsam wachsende erythematöse Knoten gekennzeichnet [3]. Das Gesamtüberleben nach kompletter Exzision von kutanen Leiomyosarkomen ist im Allgemeinen günstig, selten kommt es zu einer Fernmetastasierung [4], wobei das subkutane Leiomyosarkom eine häufigere Metastasierung aufweist als das dermale Leiomyosarkom [5]. In dedifferenzierten Leiomyosarkomen ist ebenfalls ein klinisch aggressives Verhalten mit Lokalrezidiven und möglicher Metastasierung beschrieben [6]. Eine engmaschige Nachsorge wird empfohlen, um Lokalrezidive auszuschließen, und umfasst klinische Kontrollen, insbesondere des Lokalbefundes sowie die Sonographie der regionären Lymphknoten. Staginguntersuchungen mittels Computertomographie können im Einzelfall angezeigt sein.

Der vorliegende Fall demonstriert eindrücklich, dass auch das kutane Leiomyosarkom in der Lage ist, die Schädelkalotte zu infiltrieren. Bisher wurde ein solches Verhalten für diesen Tumor noch nicht beschrieben. Die Komplexität des Verlaufes unterstreicht außerdem die Bedeutung eines interdisziplinären Therapiekonzeptes bei seltenen Tumoren.

## References

[1] Vandergriff T, Krathen RA, Orengo I (2007). Cutaneous metastasis of leiomyosarcoma. Dermatol Surg.

[2] Kohlmeyer J, Steimle-Grauer SA, Hein R (2017). Cutaneous sarcomas. J Dtsch Dermatol Ges.

[3] De Giorgi V, Scarfì F, Silvestri F, Maida P, Gori A, Trane L, Massi D (2019). Cutaneous leiomyosarcoma: a clinical, dermoscopic, pathologic case study. Exp Oncol.

[4] Schadendorf D, Haas N, Ostmeier H, Czarnetzki BM (1993). Primary leiomyosarcoma of the skin. A histological and immunohistochemical analysis. Acta Derm Venereol.

[5] Ortins-Pina A, Soares-de-Almeida L, Rütten A (2018). Primary cutaneous vascular leiomyosarcoma: A rare subtype of leiomyosarcoma of the skin. J Cutan Pathol.

[6] Chen E, O’Connell F, Fletcher CD (2011). Dedifferentiated leiomyosarcoma: clinicopathological analysis of 18 cases. Histopathology.

